# No-Fault Compensation and Anti-COVID-19 Compulsory Vaccination: The Italian Context in a Broad View

**DOI:** 10.3390/vaccines10050635

**Published:** 2022-04-19

**Authors:** Paola Frati, Nicola Di Fazio, Raffaele La Russa, Paola Santoro, Giuseppe Delogu, Vittorio Fineschi

**Affiliations:** 1 Department of Anatomical, Histological, Forensic and Orthopedic Sciences, Sapienza University of Rome, 00128 Rome, Italy; paola.frati@uniroma1.it (P.F.); nicola.difazio@uniroma1.it (N.D.F.); paola.santoro@uniroma1.it (P.S.); giuseppe.delogu@uniroma1.it (G.D.); 2 Department of Clinical and Experimental Medicine, University of Foggia, 71122 Foggia, Italy; raffaele.larussa@unifg.it

**Keywords:** COVID-19 vaccination, adverse events, no-fault compensation, consent form, information, availability, ethical issues, liability

## Abstract

Italy, like other European countries, has produced a series of regulations during the COVID-19 pandemic. Compulsory vaccination has been introduced for the Italian population. Meanwhile, the Decree-Law 27 January 2022 n. 4 provided for the compensation mechanism for those who have received damage of the psycho-physical integrity due to the anti-SARS-CoV-2 vaccination recommended by the Italian Health Authority. Law 1992 no. 210 already provided for the indemnity system for persons damaged by irreversible complications due to compulsory vaccinations, transfusions, and the administration of blood products. The legislator intended to attribute the right to an indemnity that is not compensatory in order to repair a wrong connected to some hypothesis of liability, but it rather has a welfare character in the broad sense, being attributable to Constitutional fundamentals. In the Italian panorama, although the vaccination damages have been fully included in the already existing law no. 210/1992, to date, no precise indications have been provided regarding the ascertainment of the causal link and the extent of the compensable damage. In the near future, the interest of the scientific community will focus on the evaluation of applications for access to the benefit.

## 1. Introduction

Italy, on par with the international context, following the pandemic emergency dictated by COVID-19, has issued a series of relevant regulatory provisions in the field of vaccination both in relation to their mandatory nature and with regard to any profiles of responsibility and economic refreshment. Initially, it introduced the vaccination obligation for some professional categories, such as health professionals, the law enforces, or teachers, while it recently extended it to all people over the age of 50 [1,2]. Thus, it introduced the obligation for all people of an entire age group, regardless of their job, aligning itself with other European countries, where similar measures have been announced or are about to be implemented. The only European country where the vaccination obligation for all adults, regardless of the work context, has been provided for up to this moment was Austria [3]. Those under the age of 18, pregnant women, those who have recovered from COVID-19 within the last 180 days, and people who cannot get vaccinated for health reasons are exempt from the obligation. The obligation came into effect on the 1st of February, and pecuniary penalties for unvaccinated people are provided. The mechanism adopted is, therefore, that of the indirect sanction and not the direct obligation, which would provide for the compulsory administration of the vaccine.

Greece has also provided a similar modus operandi. The Greek government has, in fact, imposed the introduction of the obligation for those over the age of 60, and those who have not been vaccinated or have not booked a vaccination are convicted and receive a EUR 100 monthly fine [4].

Both philosophers and jurists agree that restrictions on liberty can be justified if they prevent harm to others. Article 8 of the European Convention on Human Rights considers the right to physical integrity as a “qualified right”, subject to the limitation in the interests of “national security, public safety or the economic well-being of the country, for the prevention of disorder or crime, for the protection of health or morals, or for the protection of the rights and freedoms of others”.

In 2013, WHO, with its Global Vaccine Action Plan, stated that immunization should be considered the main component of the human right to health and also a responsibility of individuals, communities, and governments.

The European Court of Human Rights (ECHR) has so far avoided directly addressing the issue of the anti-COVID-19 compulsory vaccination, establishing, however, general but extensible principles concerning minors. In particular, the Court established that the interference of the public authority provided for by the law is not prohibited and that they have the nature of measures that in a democratic society are necessary for national and public security, the economic well-being of the country, the defense of order and the prevention of crimes, the protection of health or morals, and the protection of third party’s rights and freedom [5].

It follows that it is sufficient to demonstrate that the State has adopted the compulsory measure in order to protect society from disturbances caused by serious diseases, including the limitation of privacy (public safety, economic well-being of the country, disorder prevention) [6,7].

The compulsory vaccination must respond to an “urgent social need” such as the decreasing trend of spontaneous vaccinations [8], in response to the pressing need to protect public health and the health of those who cannot get vaccinated [9].

“Relevant and sufficient reasons” are needed to impose mandatory vaccination, such as the efficacy and safety of vaccines and the shared goal of achieving the highest rate of vaccination coverage [10]. It is also necessary to assess the degree of safety of vaccines in light of the considerations of the scientific community and the provision of exemption from the obligation for subjects with permanent contraindications to the vaccine [11]. The mode of coercion based on the principle of proportionality must take place through moderate penalties and not through compulsory administration and, finally, through the possibility of resorting to justice to protect one’s own rights.

The Council of Europe [12,13] has likewise invited to introduce the obligation only if supported by an objective and reasonable justification.

Similarly, the Council of State in Italy affirmed that: “the legislator, in a pandemic situation that sees the spread of an airborne virus, highly contagious and often lethal for the most vulnerable subjects due to previous diseases—e.g., cardiopathic, diabetic or cancer patients-and the elderly persons, has the duty to promote and, if necessary, impose the administration of prophylactic therapy able to prevent the disease or, at least, to avert the most serious symptoms and to stop or severely limit its contagiousness” [14].

The very recent Decree-Law 7 January 2022 n. 1 “Urgent measures to cope with the COVID-19 emergency, in particular in the workplace, in schools and higher education institutes” has established in Italy the compulsory vaccination obligation for all individuals over the age of 50 [1], while the Decree-Law 27 January 2022 n. 4 provided for the compensation mechanism for those who have received damage from the administration of the vaccine [15].

The Centers for Disease Control and Prevention (CDC) is providing timely updates on adverse events that may occur after COVID-19 vaccinations, including:

- Anaphylaxis: occurred in approximately five people per one million vaccinated in the United States;
- Thrombosis with thrombocytopenia syndrome (TTS): CDC and FDA confirmed 60 cases after Johnson & Johnson’s Janssen vaccine (more than 18.5 million doses as of 31 March 2022);
- Guillain-Barrè Syndrome: 312 preliminary reports identified in the USA as of 31 March 2022 (VAERS, Vaccine Adverse Event Reporting System);
- Myocarditis and pericarditis: 2332 preliminary reports in the USA as of 31 March 2022 (VAERS, Vaccine Adverse Event Reporting System).

## 2. Vaccination Campaign and Compensation in Italy

The Decree-Law January n. 4/2022 established to add to the previous Italian law no. 210/1992 the compensation provision to those who have suffered injuries or infirmities, from which derived a permanent impairment of the psycho-physical integrity, due to the anti-SARS-CoV-2 vaccination recommended by the Italian Health Authority. The related charge is provided pursuant to Article 32 of the Italian Constitution, which protects the health of the individual and the community and establishes the mandatory rule of health treatment only if required by law. The Italian Constitutional Court [16,17,18] had already clearly stated that the mandatory vaccination must comply with the following conditions:

- The treatment must be aimed not only at improving or preserving the state of health of cared individuals but also at preserving a third party’s state of health;
- It must not negatively affect the state of health of the person who is obliged, except for those consequences that appear normal and, therefore, tolerable;
- In the event of further damage, the payment of a fair indemnity in favor of the injured party must be provided for, regardless of the parallel compensation protection.

### 2.1. Italian Law No. 210/1992 and the Indemnity Mechanism

Law 1992 no. 210, drawing inspiration from Italian jurisprudence, provided for the indemnity system for persons damaged by irreversible complications due to compulsory vaccinations, transfusions, and the administration of blood products [19]. The common ratio is that it is not lawful to require that individual citizens expose their health to risk for a collective interest without the community itself being willing to share the weight of any negative consequences [20]. Therefore, the existence of a right has been recognized constitutionally sanctioned to compensation in the event of damage to health suffered as a result of subjecting to compulsory vaccination [21]. The original framework of Law no. 210/92 was subsequently amended and supplemented by some legislative provisions that expanded the area of protection originally envisaged [22,23,24,25].

The compensation provided for and regulated by Law 1992 no. 210 and subsequent amendments and additions are attributable to the services paid by the State for reasons of social solidarity and to testify the interest of the community in the protection of health: it deviates from any compensation for damage suffered as a result of the infection.

The legislator intended to attribute the right to an indemnity that is not compensatory in order to repair a wrong connected to some hypothesis of liability (objective or subjective) for which hospitals are responsible, but it rather has a welfare character in the broad sense, being attributable to art. 2 and 32 of the Constitution and to the services charged to the State by reason of the duty of social solidarity. It is therefore configured as an economic support measure linked to an objective situation of impairment of the state of health deriving from a health service aimed at safeguarding health itself [26].

Moreover, it is provided regardless of the economic conditions of the person entitled and can be combined with any other economic benefits received for any reason, even if due to the damage.

The recent legislation on COVID-19 related vaccination damages wanted to enclose them within the scope of application of law 210/1992, favoring the indemnity system rather than the compensation system.

### 2.2. Differences between Indemnity and Compensation

Before outlining the cases envisaged by the law for the benefit of those who have suffered damages from the adverse effects of the vaccine, it is necessary to clarify the difference between indemnity and compensation.

Compensation for damage always presupposes the existence of ascertainment of the profile of guilt, while the right to compensation is independent of it and does not require proof of an offense. Conversely, it arises solely for the verification that the irreversible impairment was caused by the vaccination. What goes beyond the right to financial compensation is the need to ascertain the profile of responsibility since this is granted on the assumption of a material connection between the administration of the vaccine and the occurrence of the event that is harmful to health [27].

Therefore, if the damage caused by the vaccine is linked to an error by the treating physician (who, for example, despite knowing a certain pathology of the patient, did not advise against the administration of the vaccine) or to the execution of the injection by the healthcare staff, or even to the administration of a vial coming from a defective batch, or in any case from other negligent behavior of one of the subjects involved in the chain of administration of the vaccine dose, it is possible to combine indemnity with compensation. In all other cases in which the adverse effects occurred following the vaccine, without any responsible person and any fault, it will still be possible to access indemnity protection, which finds its reason “in the mandatory duty of solidarity that in these cases it is incumbent on the whole community”, which benefits from the vaccination treatment of the individual.

The indemnity has an equitable nature and allows the interested parties certain protection, predefined by law [28,29].

### 2.3. Compulsory Vaccination Indemnity

The anti-COVID-19 vaccination, mandatory for healthcare professionals [30] and now for the entire population over the age of 50, is fully included in Law no. 210/1992, art. 1.

On the basis of the aforementioned rule, and according to the rules of indemnity protection, the injured party will have to prove to have suffered injuries or infirmities of such intensity as to have caused permanent impairment of psycho-physical integrity (any passing fevers or modest transient disturbances are therefore irrelevant), and that the damage suffered is a consequence of vaccination.

Personal injury ascertainment and evaluation is a pivotal issue both from a medico-legal and juridical point of view. Currently, the methodology is non-homogeneous, also due to the involvement of different professionals in the process. Evaluation criteria of injury should be intended as logical and subsequent steps, aiming to express a percentage based on the entire psychic and anatomo-functional value of the person [31].

As stated by Constitutional Court: “compensation from compulsory vaccination has constitutional coverage, since it is an economic support measure, based on the collective solidarity guaranteed to citizens, in the same way as the aforementioned art. 2 and 38 of the Constitution, in the face of events generating a situation of need [32], a measure based on the insufficiency of health checks set up in the sector” [33].

Moreover, the right to compensation has the nature of a subjective right that can be protected before the ordinary judge [34].

## 3. Vaccine Recommendation and Right to Compensation

Notwithstanding the full application of Law 1992 no. 210 to all cases in which the anti-COVID-19 vaccine has been made mandatory by means of law, it remains to be ruled about the damage that will occur to categories currently not obliged. The new legislation, in fact, refers to the mere recommendation.

The jurisprudence of the Constitutional Court had the opportunity to pronounce itself several times on the subject of recommended vaccinations [29], and recently, in the middle of the pandemic period, it did so with another judgment [35].

For the judges, there is no qualitative difference between obligation and recommendation, the common objective being that of ensuring the widest immunization, being “completely irrelevant, or indifferent, that the cooperative effect is attributable, on the active side, to an obligation or rather, to a persuasion or even, on the passive side, to the intent to avoid a sanction or, rather, to adhere to an invitation” [36]. Even if “the recommendation” always appears preferable on the political “for its” gentle “push, (which) accompanies and favors the development of self-determination, although this push also has a profound effect on the formative process of the will in informed consent, without the constraint and the extreme ratio of the obligation, it increases citizens trust in science and public intervention” [37].

The recommended vaccinations are accompanied by the promotion of extensive and repeated communication campaigns in favor of vaccination treatments, suitable for generating the trust of the population towards what is recommended by the health authorities, a fact that makes the individual choice to be vaccinated based on safeguarding not of personal interest but of the collective interest of the community. Additionally, since the individual has adhered to the vaccination due to the need of social solidarity, the State must take responsibility for any negative consequences of his psycho-physical integrity; on the other hand, it would be unfair to allow the injured individuals to bear the cost of an also collective benefit [36]. It is always the Italian judges who underline and recall what characterizes the “recommended” vaccination:

- Even extraordinary information and recommendation campaigns by the public health authorities in their highest instances;
- Gentle pushes or so-called “nudges” through a system of incentives and disincentives;
- Distribution of specific information material;
- Information contained on the institutional website of the Ministry of Health;
- Ministerial decrees and circulars;
- National vaccination prevention plans;
- The right to compensation.

The function of the indemnity, for the Italian judges, is to complete the “solidarity pact” between the individual and the community in terms of health, resulting in the need to shift to the community, the riskiness of the occurrence of the harmful effects resulting from the vaccine, so as not to infringe the rights protected by the Constitution.

### 3.1. Current International Scenario of No-Fault Compensation Programs for COVID-19 Vaccine Injury

Vaccination for COVID-19 is already in progress worldwide, and as of 10 February 2022 [38], over 10 million vaccine doses have been administered [39]. As vaccinations will continue, a great number of vaccine injury events should be expected [11].

In this light, vaccine injury compensation programs can help build confidence in vaccine safety. In the present global situation, 25 out of 194 WHO member states have implemented such no-fault vaccine injury compensation programs [40,41].

The COVAX No-Fault Compensation Program is the first international vaccine injury compensation mechanism intended for Advance Market Commitment (AMC) Eligible Economies [42,43]. This program operates in 92 low- and middle-income countries and economies, providing no-fault compensation to eligible individuals who suffer serious adverse events after receiving a COVID-19 vaccine distributed through the COVAX Facility until 30 June 2022 [44,45].

The authors identified 15 countries having vaccine injury compensation programs on four continents (USA, Canada, South Africa, Australia, China, Singapore, South Korea, UK, Austria, Belgium, France, Germany, Netherlands, Portugal, and Sweden).

#### 3.1.1. The United States of America

COVID-19 vaccine injuries are excluded from the already existing Vaccine Injury Compensation Program (VICP). Currently, all people injured by vaccines given as countermeasures during a declared emergency (such as the COVID-19 pandemic) bring claims under only the Countermeasures Injury Compensation Program (CICP). Covered countermeasures are identified by the Secretary of the Department of Health and Human Services (HHS) in declarations published under the PREP Act [46]. According to CICP data, as of 1 December 2021, the CICP has not compensated any COVID-19 countermeasures claims. Only one COVID-19 countermeasure claim, due to an anaphylactic reaction after vaccine administration, has been determined eligible for compensation and is pending a review of eligible expenses [47].

#### 3.1.2. Canada

The pan-Canadian Vaccine Injury Support Program (VISP), launched by the Canadian government in June 2021 [48], is a national vaccine injury compensation program that provides financial support to all people in Canada who have experienced a serious and permanent injury as a result of receiving a Health Canada authorized COVID-19 vaccine [49]. According to VISP, a serious and permanent injury is defined as a severe, life-threatening, or life-altering injury that may require in-person hospitalization or prolongation of existing hospitalization and results in persistent or significant disability or incapacity, or where the outcome is a congenital malformation or death.

#### 3.1.3. South Africa

As of 22 April 2021, the South African government established a COVID-19 Vaccine Injury No-Fault Compensation Scheme; its purpose is to provide easy access to compensation for persons who suffer harm, loss, or damage as a result of vaccine injury caused by the administration of a COVID-19 vaccine [50].

The National Department of Health is responsible for funding and administration of such a scheme [51]. Vaccine injuries that are covered by the scheme are severe injuries resulting in permanent or significant injury, serious harm to a person’s health, other serious damage, or death. However specific framework for such a scheme has not yet been released.

#### 3.1.4. Australia

A No-Fault COVID-19 Indemnity Scheme was released last August 2021, providing Australians with quick access to compensation for COVID-19 claims related to the administration of a Therapeutic Goods Administration approved COVID-19 vaccine [52]. The scheme will also be backdated to February 2021 [53].

#### 3.1.5. China

In China, two different programs exist, covering injuries arising from vaccines listed in the national immunization program (NIP) and non-NIP vaccines, with the government funding the first and pharmaceutical companies or market authorization holders funding the latter [54,55]. The standard of proof is based on epidemiological connection, and the regulation excludes compensation injuries that are supposed to be coincidental to vaccination.

#### 3.1.6. Singapore

The Vaccine Injury Financial Assistance Program for COVID-19 Vaccination (VIFAP) provides one-time goodwill financial assistance to persons who received the COVID-19 vaccines under the National Vaccination Program [56]. Access to financial assistance is restricted to Singapore citizens, permanent residents, or long-term pass holders who experienced serious side effects after the COVID-19 vaccination. The serious side effects must require hospitalization or cause persistent incapacity or disability, or eventually cause death. The amount of financial assistance provided is dependent on the severity of the serious side effect.

#### 3.1.7. South Korea

Similar to China, South Korea has two different programs for injuries linked to vaccines included in the national immunization program (NIP) and to those not included (non-NIP) [57]. Korean Government announced the implementation of the current system for the compensation of injury caused by the vaccination of COVID-19 [58].

#### 3.1.8. The United Kingdom

The Vaccine Damage Payment [59] is a one-off tax-free payment provided by the UK Government for citizens severely disabled owing to vaccination against different diseases, including COVID-19, as of 31 December 2020 [60]. The claim for a Vaccine Damage Payment must be made by, or on behalf of, the disabled person concerned or, if the person has died, by his/her personal representatives. Such an act is intended to compensate for “severe disability” (at least 60% disabled), which is assessed for the purposes of entitlement to disablement pension in the UK [59,60,61].

#### 3.1.9. Austria

In January 2022 Austrian Parliament declared, for the first time in Europe, COVID-19 vaccination mandatory for citizens older than 18 years old. In accordance with the Vaccination Damage Act, an Austrian citizen who experiences injuries from an obligatory vaccination or a vaccination recommended by a regulation of the Ministry of Health, is entitled to compensation [62]. COVID-19 vaccination injuries have been included in this Act.

#### 3.1.10. Belgium

COVID-19 vaccination is currently received on a voluntary basis in Belgium, but no specific system for vaccine injury compensation exists.

#### 3.1.11. France

As of 15 September, 2021, COVID-19 vaccination is mandatory for people who work with frail subjects. Concerning mandatory vaccination, the French patient can expect full compensation for any damage and should claim compensation from the National Office for Compensation of Medical Accidents (ONIAM). As defined by Decree 2021-1262 of 16 October 2020 and Decree 2021-637 of 21 May 2021 [63], a person who has suffered damage as a result of the COVID-19 vaccination is eligible for compensation [64].

#### 3.1.12. Germany

Vaccine injury compensation legislation is included in a statute that involves the control of infectious diseases, the German Infectious Diseases Protection Act [65,66,67]. The no-fault compensation program applies to compulsory vaccines and to non-compulsory vaccinations as long as the vaccination is publicly recommended.

#### 3.1.13. Netherlands

In the Netherlands, there are no mandatory vaccinations, and COVID-19 vaccination is part of the national vaccination program. The general Dutch legal system for compensation of damages, codified in the Dutch Civil Code, applies to any claim of injury caused by COVID-19 vaccines [68].

#### 3.1.14. Portugal

The National Vaccination Program (PNV) allows everyone to receive vaccines according to their age and in qualified health centers [69]. COVID-19 vaccination is voluntary, universal, and free of charge. No specific legislation governing vaccine compensation exists, and possible compensation claims are regulated by the Portuguese Civil Code and by Law 67/2007 [70].

#### 3.1.15. Sweden

On 10 November 2021, the Swedish parliament voted to approve state compensation for injuries caused by COVID-19 vaccinations [71,72]. According to this bill, the Swedish state will compensate patients with vaccination-related injuries when the Swedish Pharmaceutical Insurance, which covers anyone in Sweden, does not apply.

## 4. Conclusions

In the light of the recent SARS-Cov-2 pandemic and the introduction of compulsory or recommended vaccination for COVID-19, many states have adopted different restraint measures for the damage to health resulting from such vaccinations [73,74]. Some states have added relief for COVID-19 vaccination to measures already in place for damage resulting from other mandatory vaccinations. Others, on the other hand, have issued specific provisions in relation to vaccination against COVID-19. These measures not only aim to compensate economically those affected by serious adverse effects but also to provide the population with an incentive to vaccinate itself.

As we have already argued, “the compensation system should be simple, quick, and accessible, but above all no-fault, to provide just and equitable damages. There should be no arbitrary cap on damages. Proving causation could be facilitated by an expert-led process allowing for the identification of situations in which vaccination is linked to a particular adverse effect. Being proactive in establishing such a fund will improve the chances of any immunization program being effective while reducing overall costs to society.

The potential costs of this policy with COVID-19 vaccination remain questionable. As regards costs, previous experiences with vaccine-related injuries compensation suggests that doubts of sustainability are unsubstantiated because the costs tend to be manageable and predictable. In the meantime, more transparency in communication about compensation procedures for vaccine side effects is necessary for all those countries in which the compensation for COVID-19 vaccine-related adverse events is not in actuality foreseen” [8].

To date, there is no unambiguous assessment of the damage that can be done, as the term “serious damage to health” is often used without providing further specifications. Such non-uniformity of assessment also exposes the risk of a discrepancy between the compensation criteria of the various countries and, therefore, the actual relief granted [75]. In the Italian panorama, although the vaccination damages have been fully included in the already existing law no. 210/1992, to date, no precise indications have been provided regarding the ascertainment of the causal link and the extent of the compensable damage. Currently, citizens who believe they have suffered vaccination damage must apply to the Local Health Agency (Azienda Sanitaria Locale, A.S.L.) of competence. Subsequently, this request will be forwarded and evaluated by the relevant Hospital Medical Commission, which will express itself on the existence of the causal link, in turn forwarding the outcome of the assessments to the Ministry of Health. Since there are currently no shared guidelines, there is a high risk of valuation discrepancies and, therefore, compensation by the individual commissions [76].

In the near future, the interest of the scientific community will focus on the evaluation of applications for access to the benefit, focusing on cases in which the presence of a causal link between vaccination and the damage suffered is actually recognized [77].

## Data Availability

Not applicable.
